# Outlining the Invisible: Experiences and Perspectives Regarding Concussion Recovery, Return-to-Work, and Resource Gaps

**DOI:** 10.3390/ijerph19138204

**Published:** 2022-07-05

**Authors:** Shazya Karmali, Marie Denise Beaton, Shelina Babul

**Affiliations:** 1 British Columbia Injury Research and Prevention Unit, British Columbia Children’s Hospital Research Institute, Vancouver, BC V5Z 4H4, Canada; shazya.karmali@bcchr.ca (S.K.); denise.beaton@gov.bc.ca (M.D.B.); 2 School of Population and Public Health, University of British Columbia, Vancouver, BC V6T 1Z4, Canada; 3 Department of Pediatrics, University of British Columbia, Vancouver, BC V6T 1Z4, Canada

**Keywords:** mild traumatic brain injury, adult, workers, concussion management, concussion recovery, workplace, occupational health

## Abstract

Appropriate supports and accommodations are necessary to ensure full concussion recovery and return-to-work (RTW). This research investigated barriers and facilitators to concussion recovery and RTW, and resource gaps reported by adults with concussion (‘workers’) and workplace and healthcare professionals (‘workplaces’). Semi-structured interviews and focus groups were conducted with workers (*n* = 31) and workplaces (*n* = 16) across British Columbia. Data were analyzed using inductive content analysis. Facilitators to workers’ concussion recovery and RTW included treatment, social support, and workplace and lifestyle modifications. To address barriers, both groups recommended: (a) widespread concussion and RTW education and training (b) standardized concussion recovery guidelines; (c) changing attitudes toward concussion; (d) mental health supports; and (e) increasing awareness that every concussion is unique. Findings can inform best practice for concussion recovery and RTW among professionals in workplaces, healthcare, occupational health and safety, and workers’ compensation boards.

## 1. Introduction

In British Columbia (BC), concussions account for approximately 600 hospitalizations and 14,500 emergency department visits per year, though these are likely underestimates of the true burden [1]. Concussion, or mild traumatic brain injury (mTBI), the most common form of brain injury, is often referred to as an ‘invisible injury’, because its impacts cannot be easily observed [1]. These impacts include lasting impairments on cognitive, physical, behavioural, and emotional functioning, which can severely interfere with an individual’s ability to Return-to-Work (RTW), recreational activities, and their long-term recovery [2]. Concussion symptoms that persist for more than three months post-injury are referred to as post-concussion syndrome (PCS) and can prolong recovery and resumption of daily activities [3]. The exact incidence of PCS is uncertain due to lack of agreement on clinical and radiological criteria, and has been reported to range from 5–58% [4]. PCS may also delay an individual’s RTW strategy—a gradual, step-wise process to allow individuals to return to their employment activities safely [5]. RTW is also a measure of a successful social reintegration after trauma and an important indicator of recovery [6]. Typically, RTW following concussion occurs within two months post-concussion, but a delayed or unsuccessful RTW can have economic and psychosocial consequences for individuals with concussion as well as their families and workplaces [7].

One in six individuals have reported making changes to their work habits to manage persistent concussion symptoms at work, and one in five people report struggling to meet their employment demands over the course of months or years [8]. Some may reduce work hours or exit the workforce entirely [8]. Although adults who suffered mTBI may be able to return to their jobs post-injury, it has been reported that they may not be able to perform their pre-injury responsibilities or may not be stably employed post-concussion [9]. Using a Danish national register-based design, comparing individuals with concussion between 18–60 years old (*n* = 23,549) and matching controls (*n* = 22,228), researchers compared differences in martial stability, academic achievement, income, and socioeconomic status [10]. At 5-year follow-up, they observed a trend from gainful employment toward not working in both groups; however, this trend was significantly higher in individuals who suffered a concussion, suggesting that mTBI may be a risk factor for long-term leave due to disability [10]. In addition, fewer individuals in the injured group had transitioned from being wage earners or not working to being self-employed, leaders, or managers compared with controls—indicating the impact of concussion on occupational advancement [10]. Researchers have also reported average salary losses of 4.2% in individuals ages 20–59 who suffered a concussion; the impact of concussion on salary was observed one year post-injury and remained substantial for at least five years post-injury [11]. Another study reported that after a TBI, regardless of severity, individuals may experience long-term mental fatigue, with a higher level of fatigue reported in women and correlated with those who had a lower employment status [12]. Thus, in order to prevent decreased work participation due to persistent concussion symptoms, it is important to provide workers and workplaces with concussion education and resources [10]. This may also help reduce the likelihood of long-term work limitations and loss of productivity as a result of mTBI.

In 2017, 22% of Canadians aged 15 and over had one or more disabilities, with a higher prevalence in women (24%) and older adults (75 and older) [13]. Interestingly, females and older age groups also experience significantly more persistent concussion symptoms [14]. In individuals between the ages of 25–64, those with disabilities were less likely to be employed (59%) than those without (80%), and 76% of individuals with mild disabilities were employed, compared to 31% with severe injuries [13]. It has been reported that a longer length of disability has been predictive of poorer resumption of work and work retention [15]. This is of particular concern in the case of mild TBI, in that it has been reported to have a major impact on functioning over time—particularly regarding behavioural, cognitive, emotional, and interpersonal competencies—which also play important roles in an individual’s ability to maintain family life and employment [16,17]. As a result, researchers recommend multi-faceted rehabilitation programs for individuals returning to work post-injury [15,16]. Among those with disabilities in the 25–64 age group who were not employed and not in school, 39% had the potential to work—comprising nearly 645,000 Canadians with disabilities [13]. Therefore, ensuring systemic supports and effective RTW protocols for those suffering from conditions that prevent them from fully engaging in daily activities—such as persistent post-concussion symptoms—are crucial for re-integrating into the workforce post-injury.

Most concussion research to date focuses on youth, young adults, and sports-related concussions [18], resulting in a paucity of information and educational resources for adults [2,18]. While adults in the general population can recognize the common symptoms of concussion, such as headache and nausea, the full list of concussion symptoms (e.g., changes in mood/behaviour, difficulty sleeping) are not readily recognized [18]. The Concussion Awareness Training Tool for Workers and Workplaces (CATT WW) launched in 2019 to address this gap [19]. The CATT offers free, easily accessible, up-to-date evidence-based concussion prevention, recognition, treatment and management eLearning modules and resources for various audiences (e.g., parents, athletes, high school youth, medical professionals, and women’s support workers). The CATT WW educational modules and resources were developed with the aim of educating adults on how to prevent, recognize, and manage concussions.

CATT WW was created in partnership with WorkSafeBC to address concussion education and resource gaps for workers and their families, workplaces including employers, members of safety associations, unions, and joint occupational health and safety committees [19]. This resource was informed by direct engagement with relevant stakeholders through interviews and focus groups with adults who sustained a concussion as well as workplace and healthcare professionals who support concussion recovery and RTW. The purpose of this study is to present secondary outcomes from the CATT WW findings to determine: (a) the recovery process for adults who sustained a concussion, and their RTW experience (e.g., barriers and facilitators to returning to the workplace); (b) current concussion RTW procedures and resource gaps identified by workplace and healthcare professionals; and (c) recommendations to support Return-to-Work following a concussion reported by both workers and workplace and healthcare professionals. The findings from this research can support best practice in concussion recovery and RTW for workers, workplace and healthcare professionals, workers’ compensation boards, insurers, and family members.

## 2. Materials and Methods

As this research was conducted as part of a larger descriptive study, full methodological, recruitment, and participant demographics and injury characteristics are detailed elsewhere [19]. A brief procedural description relative to the current study is described below. This research was approved by the host institution’s Research Ethics Board (H18-00604) and by WorkSafe BC Research Services.

Using qualitative research methods, focus groups and one-on-one interviews were conducted to understand participants’ perspectives and experiences with concussion [19,20]. Participants were recruiting using purposive sampling [19]. This research was situated within a social constructivist paradigm, centered on generating meaning about an individual’s health by understanding how they experience it [21].

All interviews and focus groups were audio-recorded, de-identified, and transcribed verbatim. Transcriptions were analyzed inductively (i.e., themes came from question responses and were not pre-determined), using NVivo (Version 12) [22], by two researchers who independently reviewed responses, identified themes, discussed any discrepancies, and determined the most accurate title for each theme [20]. NVivo is a data analysis software which allows researchers to organize qualitative data, combine findings into themes and sub-themes, and sort information [22]. To ensure data trustworthiness, the researchers followed quality assurance steps during data collection and analysis such as: member checking between interview questions to ensure responses were accurately understood; reviewing, condensing, and deliberating findings to prevent bias; reflexivity; and, documenting study methods, procedures, and analyses to allow others to determine whether or not findings are transferable to other settings [23,24].

## 3. Results

### 3.1. Participant Characteristics

Thirty-one adults who experienced a concussion and returned to work or were in the recovery and RTW process at the time of the interview (hereafter referred to as ‘workers’), participated in this research. Workers were employed in various industries, including healthcare, arts, science, education, construction, transportation, administration, and security [19]. See Table S1 for detailed overview of industries in which workers were employed.

In addition, interviews and focus groups were conducted with 16 workplace and healthcare professionals, including allied health professionals, specialists, and a case manager, union advocate, physician, counselor, and office manager [19]. See Table S2 for a detailed overview of workplace and healthcare professionals’ occupations.

Interviews and focus groups with workers ranged from 14–150 min, and with workplace and healthcare professionals from 25–58 min. Saturation was reached once data collection did not yield further insights or generate new themes related to the original research questions [25]. A recent systematic review of empirically-based studies of sample sizes for saturation in qualitative research determined that saturation is typically reached after 9–17 interviews or 4–8 focus group discussions [26]. Themes and sub-themes from interviews and focus groups with workers and workplace and healthcare professionals are presented in Tables S3 and S4, respectively, and in detail with supportive quotations, below.

### 3.2. Workers’ Facilitators to Concussion Recovery and RTW

Workers described factors and accommodations that facilitated their concussion recovery and progression through their RTW program, if they engaged in one. Some workers (*n* = 4) reported that they were provided with resources during their concussion recovery that assisted them with understanding concussion symptoms and the recovery guidelines, such as brochures and information from their primary care physicians.

Participants identified the following healthcare professionals as providing significant treatments and guidance to support them through their recovery process: physiotherapist (*n* = 11); occupational therapist (*n* = 7); counselor, psychiatrist, or psychologist (*n* = 5); massage therapist (*n* = 3); neurologist (*n* = 2); sports medicine or concussion specialist (*n* = 2); vestibular therapist (*n* = 2); audiologist (*n* = 1); kinesiologist (*n* = 1); acupuncturist (*n* = 1); and physiatrist (*n* = 1). In addition, 10 participants attended concussion clinics for treatment. Participants explained the techniques that helped them and others better understand their altered abilities and capacities during recovery.

“*One of the best things [my occupational therapist] did was give me… a point system for… activity. And so that helped me talk to my family about things. … I only have this many points a day and you’re asking me to do this on top of work. … When they actually put it into terms of it’s this many points just for two people, and you have to add an extra point for each person that’s at that dinner table. And this is how many points it is for work, so this is why I’m maxing out. So, we can only do dinners on weekends. … And bringing that to work, it was easier for my boss to understand, too*.”

[“Abby”, 27, Dietician]

Social support also positively impacted workers’ recovery, not only in providing participants with companionship, but also in assisting with tasks such as grocery shopping, meal preparation, and driving. Nineteen participants indicated that they received support from their family members including parents, partners, and children. Furthermore, 13 participants noted the importance of support from peers during concussion recovery, to check in on them and/or help with activities of daily living. One participant described that her friend, a nurse, provided her with concussion recovery guidance: “*I just am so thankful I have so many good friends like that. … It was [my friend] who told me, no driving. Make sure you still see your doctor, … you can’t work. … You should be going for little walks*” [“Deanna”, 48, Clinical Nurse Educator].

A total of 23 workers explained that they experienced PCS, including: memory loss, mood and personality changes, feeling overwhelmed, anxiety/depress, confusion, and stress. Workers explained that during their recovery process, they implemented strategies that helped them cope with PCS. For instance, some participants described how they handled light and noise sensitivity resulting from their concussion by wearing sunglasses (*n* = 4) or earplugs (*n* = 3), while others would keep rooms dark or avoid noisy areas (*n* = 5). To assist with difficulties with cognition (e.g., short term memory loss, organization, and pacing themselves) three participants kept lists and notes of forthcoming tasks and plans. It was also expressed by some workers (*n* = 3) that their recovery pushed them to learn the importance of allowing themselves to take breaks and rest, as needed.

Following their concussions, participants took time off of work to facilitate recovery, which ranged from none (*n* = 5) to long-term disability or leave (*n* = 11), depending on the severity of their symptoms and recovery progress. See Table S5 for detailed overview of workers’ time off from work.

Thirteen workers required a note from their doctors for an extended period of time off work due to concussion symptoms. A total of 22 workers progressed through a graduated RTW process, in which they would attend work for fewer days and/or hours, and slowly increase as they were able. Nine workers reported that they did not partake in a graduated RTW. Characteristics of workers who did not complete a graduated RTW varied (e.g., occupation, age, etc.). Workers who reported a positive RTW experience (*n* = 12) were also among those who felt supported by both their managers and colleagues (*n* = 14) after their concussion and were permitted to implement modifications in their schedules or workplaces to facilitate their RTW. These modifications included: taking breaks throughout the day (*n* = 10); changing their work routine (e.g., working from home, using a different office space, or working shorter days; *n* = 14); environmental adaptations (e.g., light covers, blue screen on computers; *n* = 5); wearing noise cancelling headphones (*n* = 3); limiting physically or mentally demanding tasks (*n* = 8); and avoiding or reducing screen time (*n* = 2). Interestingly, only one participant reported having a positive experience with the provincial workers’ compensation board. Although some workers described their RTW process as positive due to the support they received and modifications they made, they reported that the process was overwhelming and took time to progress through. One worker described:

“*My physician and myself, we made a sort of a calendar. … We filled out, first week we’ll start with two hours [at work]. … Next week we’ll do two days at two hours. … And we just slowly increased it, and if I found it difficult at any time we went back to the [previous] week. And that’s how it was proposed to my employer. … It was probably a four-month process as a Return-to-Work for me*.”

[“Victoria”, 54, Infection Prevention and Control Practitioner]

### 3.3. Workers’ Barriers to Concussion Recovery and RTW

Workers described multiple barriers and setbacks they faced during recovery and RTW post-concussion. Eleven workers indicated that they did not receive any supplemental resources or information on concussion recovery from their healthcare providers, so they sought further information from the internet. Fifteen workers voiced that they received poor education and/or follow-up care from their physicians with respect to both their concussion in general, as well as for complex, prolonged symptoms. In addition, five workers were initially not diagnosed with a concussion, and two were misdiagnosed. One worker stated: “*Nobody actually ever said I had a concussion. Nobody ever said these are things you can do*” [“Jim”, 57, Administrative Support]. There was a common feeling that physicians themselves either may not have recognized the worker’s concussion or did not know how to treat concussion. Another worker expressed:

“*They kept telling me … you’re doing well. You’re doing well. You’re doing well. But I know I wasn’t doing well, because if I was doing so well, how come I haven’t… initiated a gradual Return-to-Work? And it’s been 16 months now as of yesterday. I just feel that that information was kind of withheld. But in truth and looking back, I think they didn’t even know*.”

[“Daphne”, 50, Nursing Assistant]

They also described a general lack of addressing mental health post-concussion, as well as poor availability of mental health resources targeted towards concussion recovery. Three workers reported that their physicians prescribed them medication instead of addressing the underlying causes of their symptoms.

Two workers noted that they did not have any family support, while six workers expressed that while they may have initially felt supported by their family and friends, their family and friends did not fully understand prolonged symptoms of concussion. One worker explained the impacts of her concussion on her social life and her family: “*My family has suffered because of what’s going on. My social life is basically nothing. Nobody really wants to talk to me. I’m also draining a lot of people that I speak with because I’m trying to decompress, but I’m becoming quite needy as a person with my friends. … My relationship is on the rocks now because financially it’s taken a real hit for us to get this far—to access therapies, which [my insurer] won’t pay for or didn’t want to pay for. I’m disruptive at nighttime. So, my husband doesn’t sleep very well”* [“Daphne”, 50, Nursing Assistant].

Several workers described a general lack of understanding, support, and empathy from their insurers or workers’ compensation board (*n* = 16), managers (*n* = 7), colleagues (*n* = 3), and employers (*n* = 2) during their recovery and RTW. They experienced increased stress because their workers’ compensation board or insurance companies did not believe the magnitude of their prolonged concussion symptoms, which resulted in claims being closed or dismissed, and wages being cut off. Workers who were removed from their benefit programs were forced to use their sick time for their concussion recovery. The culmination of these experiences heightened financial stress and resulted in some individuals applying for employment insurance or returning to work before they were ready. Workers explained that because of the invisible nature of this injury, their peers assumed they were healed while others forgot that they were recovering from a concussion. Five workers reported a negative graduated RTW experience because their workplaces, managers, or colleagues assumed they were able to take on a full work load, did not implement accommodations, or did not connect with them to assess their progress. Some reported that the lack of support from, or fear of being viewed negatively by, their employers made them hesitant to ask for workplace accommodations.

Importantly, workers described that concussion recovery accommodations vary based on occupation and industry, and a lack of awareness of these differences can make concussion recovery even more difficult. For instance, stunt performers in the television and film industry (*n* = 7) described 12-h work days and physically demanding jobs, thus directors would need to consider significantly shorter work days and tasks that do not put workers at risk of another injury. In healthcare settings, workers explained struggling with fluorescent lighting, loud and busy work environments, and in education settings, teachers struggled with visual stimuli, needing extra time to set up classrooms, and the inability to take frequent breaks. One teacher explained: “*Teachers have other challenges that other workplaces don’t. They need accommodations to their supervision duties, possibly even their positions (e.g., a PE teacher may need to be placed in a classroom while they are recovering), location of classroom(s) to minimize travel through challenging and unsafe areas (e.g., not having to cross playground areas to their classroom where they may get hit with a ball), work load accommodations-assignment of subjects/grades that they are familiar with, support for students in their classes who may be verbally and physically aggressive, as this is putting the employee with an mTBI at risk of re-injury”* [“Mayra”, 45, Teacher].

Lastly, 12 workers described administrative barriers to their concussion recovery. They noted that it was difficult to locate the correct forms required for a workers’ compensation claim, and that the process was unclear, particularly given that they were struggling with a brain injury, thus severely impacting their ability to comprehend the forms and the procedure for submitting them.

### 3.4. Current Concussion RTW Procedures and Gaps Identified by Workplace and Healthcare Professionals

#### 3.4.1. Current Concussion RTW Procedures

The workplace and healthcare professionals interviewed for this study support workers who are progressing through a graduated RTW process in a variety of ways. Those who worked for insurers or the workers’ compensation board (*n* = 3) ensure adults with concussion are accessing treatments for RTW, advocate on behalf of the worker by corresponding with employers regarding workplace accommodations, process appeals to claims decisions, and review complaints. One workplace professional described that his role involves assessing injury legitimacy by investigating whether the mechanism could conceivably cause an injury, and speaking with witnesses to verify the incident. In addition, workplace professionals described that they often try to meet with workers with a concussion in person, as well as with employers and human resources administrators, to ensure they thoroughly understand the severity of the worker’s injury and their employer’s expectations for a RTW process. However, two workplace professionals noted the difficulty of entering a worker’s place of employment as they are often met with resistance from employers and human resources departments.

Two healthcare professionals explained that they assist workers with concussion with their RTW by generating the plan, assessing workplaces for environmental accommodations, designing exercise programs, and conducting a work task analysis. Others (*n* = 5) provided workers with resources that describe concussion recovery, such as websites or brochures.

#### 3.4.2. Workplace Gaps

Workplace and healthcare professionals identified many gaps that create challenges when assisting with concussion recovery and RTW. Some professionals described systemic issues such as inability to reach rural populations (*n* = 2), lack of a standardized process in addressing concussion (*n* = 3), and lack of resource to support concussion recovery that does not follow a ‘typical’ trajectory (*n* = 5). It was noted that there is no pathway to follow or milestones to check for when assessing a worker’s case, thus making it more challenging to support a worker in their recovery. A workplace professional expressed: “*[For other injuries] I can talk with other medical advisors. … I can verify certain things and I can dispute certain things. There’s nothing like that with concussions. We don’t have protocols and checks and balance in place … I don’t have any mechanism to accurately determine what a person is saying to me and saying does this make sense for this injury or is it something else*” [“Kevin”, Insurance Case Manager]. They expressed that case managers and workers’ compensation board representatives should be involved in concussion recovery and RTW as early as possible, but workers are currently expected to advocate for themselves and left feeling as though they must convince anyone involved in their claim that they are, in fact, injured. They explained:

“*When someone has an injury, they first need to convince their employers they have an injury. And then they need to convince their doctors they have an injury. And then they need to convince the entitlement officer they have an injury. And by the time it gets to a case manager whether there actually was an injury or not, now they have one. … By the time it comes to me often I’m dealing with the psychological aspects and the emotional aspects and the belief that something is terribly wrong. Whether there’s actually something terribly wrong or not*.”

[“Kevin”, Insurance Case Manager]

A few workplace professionals interviewed noted that they are only integrated into the RTW and recovery process approximately three or more months post-concussion. At this point, almost all workers they encounter are experiencing PCS.

Ten workplace and healthcare professionals expressed that there is a lack of concussion knowledge among insurers and workers’ compensation boards. They explained that these organizations believe that every concussion follows the same recovery trajectory, and that workers should be back at work within approximately six weeks post-concussion. There is a lack of awareness and understanding that no two concussions are the same, and that recovery varies by individual, potentially with prolonged PCS. As one healthcare professional describes: “*[Insurers] don’t have understanding of concussion injury and how impacting it can be on a person’s life. … So, they expect people to be back at full-time work within six weeks of their RTW plan starting. So, they tend to have more strict guidelines, so you have to really justify why it might take longer for some people*” [“Zoe”, Occupational Therapist].

Nine workplace and healthcare professionals identified that this lack of concussion knowledge also exists among employers and workers. They further explained that because concussion is an invisible injury, many employers or colleagues do not understand challenges a worker with concussion may be facing as they do not appear to be noticeably impacted. Moreover, eight respondents highlighted that workers with concussion themselves often do not understand concussion recovery, persistent symptoms, and graduated RTW, and many believe that they need to be fully healed before resuming even adapted versions of their work and usual activities. It was highlighted that workers are often not aware of available concussion supports or resources in the community. There was a consensus that lack of standardized training on concussion recovery for workers and workplace or healthcare professionals poses a significant challenge when addressing concussion recovery and RTW.

A further concern outlined by workplace and healthcare professionals (*n* = 12) was an absence of information on, and treatment for, mental health following a concussion. For instance, workers with concussion were not aware that they could develop anxiety or depression after a concussion. One workplace professional explained that, in their experience, mental health conditions resulting from concussion are typically difficult to cover in workers’ compensation claims.

### 3.5. Recommendations for RTW

#### 3.5.1. Workers

Fifteen workers expressed the need for wider ranging patient resources following their concussion. It would have been beneficial to have a patient advocate who could help with administrative tasks such as navigating insurance applications. Others described that an external advocate with concussion training could have closely observed their progress during their RTW and decided on whether or not they should continue or take a break. For instance, three film and television industry workers explained that the first aid attendant on many of their sets is the same person responsible for craft services. Thus, having a trained, external individual—who has knowledge on concussion and whose opinion is respected by workplace professionals—in positions of authority to provide expert advice would alleviate stress and anxiety on workers who may be struggling through symptoms to continue working. It was also mentioned that having access to someone who was aware of specific concussion resources and community supports for different industries or professions would be valuable.

Overwhelmingly, workers (*n* = 25) expressed the need for wider reaching concussion awareness, education, and training for themselves and those around them. They explained that it would be valuable to have had knowledge on concussion and its potential long-term impacts in order to understand and aid their own recovery. Additionally, they wanted their workplaces, employers, colleagues, workers’ compensation boards, insurers, and family caregivers to know that: (1) concussion can happen anywhere and to anyone; (2) it is not just sports-related; (3) the impacts are long-term and can be severe; (4) concussion impacts everyone differently; and (5) recovery takes energy, time, and patience. They emphasized that individuals in all industries working at all levels should engage in continuous education on concussion prevention, recognition, management, and recovery. One worker explained they would want their workplace and colleagues to understand that:

***“****[Concussion is] more than just headaches and short-term memory loss and sensitivity to light. Like the depth that it can get to so you recognize that and take it more seriously off the bat. How to manage it. Prevention of it ever occurring in the first place. Like we discussed having everyone educated so that we’re not in these positions where [we are] repeating unnecessary damage to the brain. And then support for after someone’s been going through it and… they’ve figured out how to manage and they’re working on their recovery*.”

[“Y”, 26, Stunt Performer]

Others hoped that an increase in concussion awareness and knowledge would change the culture and attitudes toward concussion to increase prevention, encourage reporting, and improve understanding of the impact of persistent symptoms. One worker explains:

**“***One guy … was doing a hand pull, so someone else pulling him. … Went into the wall. He was apparently convulsing on the ground. And so, the [assistant director] watched the replay. They come over and they say, “Okay, we need to go again.” … And this guy shakes it off and the first aid attendant [who also provides craft services] there was going… “I don’t think he should go.” And this person’s like, “yeah, I guess I’m okay.” And, great, we’re going again. And so does the same thing. Hits his head again. … This guy is throwing up in the garbage can with the first aid attendant there. [Assistant director] said, “We didn’t get it. Can you go again?*”

[“L”, 51, Stunt Performer]

Two workers explained that lack of understanding and support made them feel lonely and isolated, and many workers (*n* = 11) identified the need for mental health and emotional support during concussion recovery and RTW.

Lastly, four workers voiced the desire for standardized concussion care. They expressed a need for a standard response when a concussion is recognized and diagnosed, and that healthcare professionals should be providing patients with consistent concussion messaging. In the same way that a visible injury is treated in a standardized manner by healthcare professionals, concussion should have the same response and support. This, in turn, would aid in patient understanding and an accelerated recovery.

#### 3.5.2. Workplaces

The majority of workplace and healthcare professionals (*n* = 12) stated the need for increased education on concussion recovery for workers who sustain a concussion, their workplaces and colleagues, workers’ compensation board, insurers, and family caregivers. They felt workers need resources and ongoing training to better understand their own concussion recovery and RTW guidelines. They stressed the importance of reminding all stakeholders (e.g., case managers, occupational experts, physicians, rehabilitation staff, advocates, employers, community members) that every concussion is different, and recovery is multifactorial and non-linear. As one workplace professional expresses: “*We all [need to] come to a common understanding [that] concussions are real. Secondary conditions are real. The mental health conditions that may arise are real. Here are the impediments to Return-to Work for the accepted and the non-accepted conditions. Here are the resources that are available… So, we’re all on the same page*” [“Bruce”, Workers’ Union Advocate]. They noted that employers and colleagues need increased awareness on differing workplace accommodations, depending on the worker’s industry and role, and tools or training on how to interact with a worker who has sustained a concussion. The recommendation was made to create a more cohesive collaboration between the worker’s compensation board and employers to seamlessly integrate workplace accommodations and support RTW. One healthcare professional explained:

“*Return-to-Work with concussion can take a lot of time. It’s not a cookie-cut approach. It has to be fluid and move with the person and how their symptoms are responding with the workload. I think the other big piece as well is that a gradual Return-to-Work after concussion is considered the most successful way to get people back to work. It helps them to be productive members of society early on which actually can help with their recovery if they feel like they’re giving back*.”

[“Zoe”, Occupational Therapist]

Seven workplace and healthcare professionals noted that resources for family caregivers should explain that adults with concussion are encouraged to resume activities of daily living, as they are able, and that these resources should be available in multiple languages and culture-specific.

Three workplace and healthcare professionals (expressed the need to standardize concussion care and messaging. They explained that they would like to refer adults with concussion to standard, effective, evidence-based resources and treatment programs for concussion management and recovery. A recovery pathway with treatment options was requested, in which they could direct patients through treatments or protocols as opposed to having to guess what to do if a certain treatment is not effective. It was suggested that concussion should be normalized in order to help increase awareness and encourage reporting. As one workplace professional reflects:

“*How do we normalize having a concussion? And what is the normal trajectory? How do we develop empathetic but firm language around what we expect as far as people’s recovery? How do we reassure people? And then… beyond that how do we educate our physicians in the community so that we’re all speaking the same language? That we’re all saying the same things*.”

[“Dolores”, Insurance Case Manager]

## 4. Discussion

This research is part of a larger, descriptive study aimed at understanding concussion recognition, diagnosis, treatment, recovery, and RTW among adults with concussion and workplace and healthcare professionals. The aim of this study was to determine the recovery process for adults who sustained a concussion as well as their RTW experience, including barriers and facilitators; current practices and resource RTW gaps reported by workplaces; and, recommendations provided by both workers and workplace and healthcare professionals with respect to RTW following a concussion. Workers described the various workplace accommodations they were able to implement to support RTW, which is line with those described by other researchers. Gordeau, Fingold, Colantonio, Mansfield, & Stergiou-Kita [27] assessed workplace accommodations following concussion, and found that common modifications included changes to work duties, modified hours, taking frequent breaks, environmental modifications (e.g., moving workspaces due to lighting or noise), and methods to assist with memory, attention, or concentration. In addition, they reported that the majority of their respondents engaged in gradual RTW [27]. Workers in the present study also identified important RTW considerations when working with individuals from various industries, and that those accommodations will vary depending on work environment and tasks.

Workers explained facilitators and barriers during their RTW following their concussions. They noted the significance of having support from employers, colleagues, and family and/or friends throughout their concussion recovery and RTW, which in turn allowed them to have a positive experience Conversely, not feeling supported by colleagues, supervisors, insurers, or workers’ compensation board resulted in a negative RTW experience for workers and increased stress, which further exacerbated symptoms. Graff, Deleu, Christiansen, & Rytter [28] investigated facilitators and barriers to RTW after mTBI and reported that the worker-employer relationship can act as both a facilitator or barrier to concussion recovery and RTW. The researchers highlight similar findings to the present study in which barriers included workers feeling obligated to return to work sooner and increase hours despite worsening of symptoms, while facilitators included supportive and understanding co-workers [28]. They also found that employers displayed a lack of awareness and knowledge of the impact of concussion on workers [28]. The experience of workers having to advocate for themselves during RTW, and challenges in communication with insurers resulting in a delay in RTW has been reported by other researchers [29].

Interestingly, workers in the current study reported receiving inadequate education from, and follow-ups with, physicians as well as a general lack of supports or resources with respect to mental health outcomes post-concussion. Mental health disorders (e.g., depression), which can occur as a result of persistent post-concussive symptoms, are often difficult to detect in that they are usually exacerbated by real-world settings (e.g., work environment) that are hard to replicate in a clinical setting [17]. In a qualitative study consisting of semi-structured interviews with 11 family physicians to understand barriers and facilitators to address mental health complications following mTBI in primary care, it was noted that family physicians generally perceived themselves as having an important role in post-concussion care specifically related to screening for mental health problems, providing education about concussion, and coordinating further care [30]. Family physicians in the cited study were generally not aware of evidence-based guidelines or tools to guide their practice regarding mental health treatment post-concussion and had trouble referring patients to specialists due to lack of a standardized process [30]. Thus, further education, screening tools, and supports for physicians to aid in mental health outcomes post-concussion would be valuable for the RTW and recovery process.

Engaging workers, healthcare, workplace, and occupational health and safety professionals allowed the researchers to compare post-concussion recovery and RTW recommendations from various perspectives. Both groups recommended: (a) more information and resources for adults with concussion; (b) increasing awareness and education of concussion prevention, treatment, management, recovery, and RTW for family caregivers, employers, colleagues, workers’ compensation boards, and insurers; (c) standardized, evidence-based concussion recovery guidelines across healthcare professionals; (d) changing the culture and attitudes toward concussion to normalize it; (e) mental health supports post-concussion, and (f) ensuring messaging around concussion emphasizes that every concussion is different and that recovery is individual and takes time.

Gaps during RTW and recovery reported by participants in this study are similar to those outlined by other researchers, for instance: (a) better collaboration between employers and healthcare professionals during RTW; (b) access to qualified professional expertise and interdisciplinary care; (c) need for social support and employer support; and (d) better awareness of mental, physical, and cognitive consequences of a TBI [31,32]. Researchers have recommended that post-TBI recovery programs should include more comprehensive considerations for the following groups: older patients; individuals with mild/moderate TBI; and individuals in the acute (0–6 weeks) and sub-acute (7 weeks–3 months) phases of rehabilitation [31]. It has also been suggested that there is a need for further research on RTW and activities of daily living post-TBI, the role of families and social networks, peer support, and continuity of post-concussion care [31]. A study conducted in the United Kingdom comparing early, specialist-led vocational rehabilitation plus usual care, compared with usual care alone, on work retention 12 months post-TBI found that RTW was most strongly related to social participation and work self-efficacy, and vocational rehabilitation was most successful when implemented early post-injury [33]. It was also reported that the parameters of what encompasses a successful RTW varies, depending on the stakeholder. For instance, for employers, prioritizing communication between employer, employee, and rehabilitation services, and understanding the impact of TBI on productivity would result in a successful RTW process [33]. On the other hand, workers who experienced TBI prioritized self-confidence, understanding their brain function and the impact of TBI on daily life activities, and needing all relevant parties to understand the effects of TBI on the individual [33]. Future RTW interventions should incorporate collaborative approaches wherein all stakeholders in concussion recovery and RTW are involved.

Findings from this research informed the development of the CATT WW eLearning module (https://cattonline.com/workers-workplaces/, accessed on 10 May 2022) which incorporates knowledge gaps that were identified from the focus groups and one-on-one interviews, and provides in-depth education on concussion prevention, recognition, response, and managing RTW and persistent symptoms. This resource also provides additional evidence-based tools and information on concussion and concussion recovery. Integrating resources such as the CATT WW into the workplace would be valuable to workers, workplace and healthcare professionals, and workers’ compensation boards, in that it would ensure that relevant stakeholders understand the impacts of concussion and how best to support an effective RTW. Moreover, specialized, interdisciplinary care has been shown to significantly reduce persistent concussion symptoms, including reduction in mental fatigue, improved social functioning, and higher satisfaction with working conditions and leisure activities [34]. Thus, allied health professionals play a unique and important role in guiding individuals through concussion recovery and RTW.

Researchers have reported on the long-term implications of extended time off of work due to a concussion. Theadom and colleagues [8] explored employment status, work limitations, and productivity loss four years after mTBI, in New Zealand. They found that more than half of adults with concussion experience challenges during their RTW, and that individuals who sustained mTBI reported far greater difficulties at work than the New Zealand general population four years post-injury. They also reported a work productivity loss of 3.6%, four years after mTBI, which was higher than the New Zealand population norm of 2.3%. Fallesen and Campos [11] studied the socioeconomic effects of concussion on working age adults in Denmark and discovered that adults (20–59 years old) who suffered a concussion over the study period experienced average salary losses of 4.2%. They also found that, over a five-year period, total income decline was lower than salary decline, potentially due to the impact of concussion on salary results from individuals exiting the labour force and collecting income from avenues such as early retirement, disability pension, or self-sufficiency [11]. Other researchers have reported that some workers continue to rely on wage replacement benefits two years post-TBI [29]. Given that 35.5% (*n* = 11) of workers in the present study reported that they were on long-term leave or disability, it is important to ensure that stakeholders have adequate knowledge and appropriate tools to address concussion early on and facilitate return to normal activities, as soon as it is safe to do so.

While the present research allowed for exploration of experiences and perspectives of workers and workplace and healthcare professionals from a variety of industries, the sample was homogenous in that the majority of worker and workplace participants were primarily English-speaking, female, middle-aged, urban-dwelling, and securely employed. An understanding of family caregivers, insurers, managers, and employers’ experiences with supporting concussion recovery and RTW may further aid in developing concussion tools and education and addressing gaps when supporting an adult with concussion. Although findings from the current research cannot be completely generalized to all adults who experience concussion, they are in line with findings from other researchers, which strengthens the rationale for developing the CATT WW, and for continuing to advocate for further education and resources for all stakeholders involved in concussion recovery and RTW.

## 5. Conclusions

The findings from this research have important implications for the future of concussion recovery and implementation of RTW protocols, by informing healthcare professionals, occupational health and safety committees, and workplaces to better understand how to prevent, recognize, and support the recovery and RTW process among adults with concussion. Both workers and workplace and healthcare professionals recommend increasing concussion awareness among adults with concussion, family caregivers, employers, colleagues, workers’ compensation boards, and insurers; addressing mental health concerns post-concussion; and standardizing concussion care. Most of all, they stress the importance of understanding concussions can happen to anyone, and though the injury is invisible, the impacts are real, and recovery requires energy, patience, and time.

## Data Availability

These data presented in this study are available on request from the corresponding author. These data are not publicly available due to privacy.

## Supplementary Materials

The following supporting information can be downloaded at: https://www.mdpi.com/article/10.3390/ijerph19138204/s1.
