# Supplementary material for: Outlining the Invisible: Experiences and Perspectives Regarding Concussion Recovery, Return-to-Work, and Resource Gaps
Source: Int J Environ Res Public Health. 2022 Jul 5;19(13):8204. doi: 10.3390/ijerph19138204 (PMC9266414; doi:10.3390/ijerph19138204)
Supplement: Supplementary file 1 [file ijerph-19-08204-s001.zip › ijerph-1744760-supplementary.pdf]

Table S1. Industries in which Workers were employed

| Workers' Industry | n (%) |
|---|---|
| Healthcare | 15 (48.4) |
| Arts, entertainment, and recreation | 7 (22.6) |
| Scientific/technical services | 3 (9.7) |
| K-12 education | 2 (6.5) |
| Construction | 1 (3.2) |
| Transportation and warehousing | 1 (3.2) |
| Administrative support | 1 (3.2) |
| Security | 1 (3.2) |

Table S2. Workplace and Healthcare Professionals' Occupations.

| Workplace and Healthcare Professional Occupations | n (%) |
|---|---|
| Workplace professionals | |
| WorkSafeBC case manager | 2 (12.5) |
| Workers union advocate | 1 (6.3) |
| Healthcare professionals (employed at concussion clinic) | |
| Occupational therapist | 5 (31.3) |
| Kinesiologist | 2 (12.5) |
| Psychologist | 1 (6.3) |
| Physician | 1 (6.3) |
| Neuropsychologist | 1 (6.3) |
| Physiotherapist | 1 (6.3) |
| Clinical counselor | 1 (6.3) |
| Office manager | 1 (6.3) |

Table S3. Themes and sub-themes extracted from Worker interviews

| Overarching Theme | Theme | Sub-Theme | Sub-Theme |
|---|---|---|---|
| 1. Facilitators to concussion recovery and RTW | | | |
| | A) Modifications | A1) Modifications for concussion recovery | A2) Modifications for RTW |
| | B) Supportive managers and colleagues | | |
| | C) Patient resources | | |
| | D) Access to healthcare professionals | D1) Techniques to help them cope with and understand their concussions | |
| | E) Social support | E1) Family support | E2) Peer support |
| | F) Strategies to cope with PCS | | |
| 2. Time off work | | | |
| 3. Barriers to concussion recovery and RTW | | | |
| | A) Administrative barriers | A1) Locating forms, filing claims | |
| | B) Barriers related to accessing information | B1) Had to find own information | B2) Not provided with any extra information or resources |
| | C) Physician-related barriers | C1) Felt physicians did not provide enough information/treatment for their concussion or persistent symptoms | C2) Challenges related to diagnosis and treatment: undiagnosed, misdiagnosed |
| | D) Lack of mental health resources post-concussion | D1) Physicians prescribed medication instead of treating underlying issue | |

| | | | |
|---|---|---|---|
| | E) Lack of social support or understanding from support networks | | |
| | F) Lack of understanding from workers' compensation boards and insurers | F1) Difficulty with claims, suspicion from insurers | |
| | G) Lack of understanding from workplaces: employers, managers, colleagues | G1) Lack of awareness about modifications/accommodations post-concussion; industry-specific modifications | G2) Lack of awareness of long-term impacts of concussion and concussion recovery |
| 4. Recommendations to support concussion recovery and RTW | | | |
| | A) Improve concussion resources | A1) Better resources for patients recovering from concussion (e.g., advocate) | |
| | B) Wider reaching concussion awareness, education, and training at all levels | B1) Training and education to increase understanding and empathy with respect to concussion recovery for workplaces, insurers, workers' compensation boards, and families/caregivers | B2) Increase awareness of limitations due to concussion symptoms and long-term effects of concussion |
| | C) Mental health resources for concussion recovery | | |
| | D) Standardize concussion care | | |

Table S4. Themes and sub-themes extracted from Workplace and Healthcare Professionals interviews

| Overarching Theme | Theme | Sub-Theme | Sub-Theme |
|---|---|---|---|
| 1. Current RTW practices/procedures | | | |
| | A) Advocating for patients/clients | | |
| | B) Administrative | B1) Intake assessments, injury legitimacy, processing claims and complaints | |
| | C) Generating RTW plan | C1) Difficulty collaborating with workplaces | |
| | D) Provide patients/clients with concussion information | | |
| 2. Workplace gaps | | | |
| | A) Systemic issues | A1) Access to all populations (e.g., rural) | A2) No resources on how to treat different concussion 'trajectories'; no standardized response |
| | B) Lag in time from injury to involvement in case | | |
| | C) Lack of knowledge among insurers and workers' compensation boards | C1) Believe every concussion follows the same recovery pattern; lack of understanding | |
| | D) Lack of understanding of concussion among the individual with the concussion | | |
| | E) Lack of mental health information/resources post-concussion | | |

| | | | |
|---|---|---|---|
| 3. Recommendations to support concussion recovery and RTW | | | |
| | A) Increased concussion education for all individuals involved in concussion recovery, including the individual themselves | A1) Every concussion is different | |
| | B) Increased awareness on accommodations/workplace modifications post-concussion | B1) Industry-specific recommendations/guidelines | B2) Collaboration between stakeholders involved in RTW |
| | C) Better resources for families/caregivers | | |
| | D) Standardize concussion care and messaging | | |

**Table S5.** Length of Time Workers were off Work Post-Concussion.

| Length of Time | n (%) |
|---|---|
| No time off | 5 (16.1) |
| Returned to work after 1-2 days | 1 (3.2) |
| Returned to work after 1 week | 3 (9.7) |
| Returned to work after 2 weeks | 1 (3.2) |
| Returned to work after 3 weeks | 2 (6.5) |
| Returned to work after 4 weeks | 2 (6.5) |
| Returned to work after 12 weeks | 4 (12.9) |
| Long-term disability or leave | 11 (35.5) |
| Unspecified time off work | 2 (6.5) |
